# Supplementary material for: The common personal behavior and preventive measures among 42 uninfected travelers from the Hubei province, China during COVID-19 outbreak: a cross-sectional survey in Macao SAR, China
Source: PeerJ. 2020 Jun 19;8:e9428. doi: 10.7717/peerj.9428 (PMC7307564; doi:10.7717/peerj.9428)
Supplement: Supplemental Information 2 [file peerj-08-9428-s002.pdf]

返澳滯留湖北人士問卷調查

年/月/日

A. 個人資料

姓名：________________ 性別：□ 男 □ 女 出生日期：________________

國籍：______________ 電話：______________________ 職業：_____________________

教育程度： □ 小學 □ 中學 □大學學士 □碩士或以上

湖北地址：____

B. 個人健康狀況及生活習慣

1. 你認為這次沒有受感染的主要原因是（可多選）:
   - □ 個人身體好
   - □ 個人防護措施好
   - □ 遠離人群/減少群集
   - □ 社區相對安全
   - □ 飲食生活習慣（均衡飲食、注重鍛鍊）

2. 在湖北疫情期間是否有規律地服用藥物/保健品作預防疫症用途？
   - □ 有，請勾選：
     - □ 西藥，請列出藥名：____
     - □ 中藥，請列出藥名：____
     - □ 保健品（營養補充/維他命等），請列出品名：_______________________
   - □ 沒有

3. 在湖北期間心情如何？

   □ 緊張 □ 焦慮 □擔心 □平淡

   如有其他請列出 :____

4. 你是否有罹患慢性病嗎？
   - □ 有，請勾選所罹患之慢性病：
     - □ 高血壓 □ 冠心病 □ 糖尿病 □ 高血脂 □痛風/高尿酸
     - □ 腦血管病 (中風) □ 慢性腎病 □ 肝炎 □ 肝硬化
     - □ 慢性阻塞性肺病 □ 其他請列出：______________________________
   - □ 沒有

5. 請問是否用長期使用西藥的習慣？
   - □ 有，請列出：____
   - □ 沒有

6. 請問是否用長期使用中藥的習慣？

☐ 有，請列出：____________________________________

☐ 沒有

7. 平常生病時你會偏好服用中藥或西藥？

□ 中藥　　□ 西藥

8. 在湖北疫情期間，請就以下健康促進行為的狀況勾選：

運動：	□ 比之前少	□ 沒有改變	□ 比之前多
睡眠充足：	□ 比之前少	□ 沒有改變	□ 比之前多
多喝水：	□ 比之前少	□ 沒有改變	□ 比之前多
買口罩和清潔用品：	□ 比之前少	□ 沒有改變	□ 比之前多
洗手：	□ 比之前少	□ 沒有改變	□ 比之前多
搜尋健康相關資訊：	□ 比之前少	□ 沒有改變	□ 比之前多

9. 在湖北疫情期間是否曾出現以下症狀：

□ 發熱	開始: _____	結束_____		□ 咳嗽	開始: _____	結束_____	
□ 乏力	開始: _____	結束_____		□ 氣促	開始: _____	結束_____	
□ 咽痛	開始: _____	結束_____		□ 流涕	開始: _____	結束_____	
□ 肺炎	開始: _____	結束_____		□ 腹痛	開始: _____	結束_____	
□ 嘔吐	開始: _____	結束_____		□ 腹瀉	開始: _____	結束_____	

如有其他請列出 :____________________________________

10. 你有經常體力活動(包括工作、家務和運動)嗎 ？

☐ 打掃、清潔等家務、步行

平均每周: □少於 1 小時 □1-3 小時 □大於 3 小時

☐ 慢跑、騎自行車、太極

平均每周: □少於 1 小時 □1-3 小時 □大於 3 小時

☐ 球類等競爭性運動

平均每周: □少於 1 小時 □1-3 小時 □大於 3 小時

☐ 沒有

11. 你是否有吸煙的習慣 ？ □有 □已戒煙 □從不吸煙

12. 你是否有酗酒的習慣 ？ □有 □沒有

C. 在湖北具體行程

1. 是否長期在湖北居住？ □是 □否

   如否，湖北停留日期：____________至________________

   前往湖北目的： □ 探親 □ 公幹 □其他 _______________

2. 居住的地方屬：

   □ 賓館 □ 居家 □ 出租屋

3. 居住的地方位於:

   □ 市中心 □ 市郊或小鎮 □ 農村

4. 居住地方之同居人數：_________ 人

5. 居住地方之面積：__________________平方尺

6. 共多少人使用同一寢室： □ 自己 □ 2 人 □ 3 人 □ 4 人或以上

7. 除填寫住址的市外，是否有前往湖北省內其他市？ □有 □沒有

   如有，請列出：

   i. 市鎮名稱__________________ 停留日數__________________

   目的__________________ 前往方式__________________

   ii. 市鎮名稱__________________ 停留日數__________________

   目的__________________ 前往方式__________________

   iii. 市鎮名稱__________________ 停留日數__________________

   目的__________________ 前往方式__________________

8. 在湖北逗留期間，是否有前往湖北以外的省份？ □有 □沒有

   如有，請列出：

   i. 省份名稱__________________ 停留日數__________________

   目的__________________ 前往方式__________________

   ii. 省份名稱__________________ 停留日數__________________

   目的__________________ 前往方式__________________

   iii. 省份名稱__________________ 停留日數__________________

   目的__________________ 前往方式__________________

9. 在湖北期間的日常生活：

   □ 自行購菜 □ 隔離小區送

10. 在湖北期間有無接到門診電話？ □ 有 □沒有

D. 接觸史

1. 在湖北期間，你主要接觸的人員是：

   □ 家人 □ 朋友 □ 工作夥伴 □ 其他__________________________

2. 在湖北期間，你是否曾前往醫院或診所？ □有 □沒有

   如有，前往醫院或診所的原因：______________________________

   日期及逗留時間：____________________________________

3. 在湖北期間，你是否曾入住醫院？ □有 □沒有

   如有，住院原因，醫院名稱及逗留期間：______________________________

   住院期間主要接觸的人員 □ 家人 □ 朋友 □ 工作人員

4. 在湖北期間，你是否有接觸新型冠狀病毒確診或疑似患者？ □有 □沒有

   如有，接觸方式(例如：吃飯)：____________________________

   接觸時間：□10 分鐘以下 □10 至 30 分鐘 □30 分鐘以上

5. 在湖北期間，同住家人是否有接觸病人/新型冠狀病毒確診或懷疑患者？

   □有 □沒有

   如有，接觸方式(例如：吃飯)：____________________________

   接觸時間：□10 分鐘以下 □10 至 30 分鐘 □30 分鐘以上

6. 在湖北居住/曾逗留的小區中，是否有新型冠狀病毒確診患者？ □有 □沒有

   如有，具體描述: ____

7. 由 1 月 1 日至”封城”，是否:

   乘搭公共交通工具 □沒有 □有 具體:

   到超級市場 日期___________ 停留期間 ___________________

   到傳統市場（濕街市）日期___________ 停留期間 ___________________

   到其他人多場合

   _________________________日期 _______ 停留期間 ___________________

________________________日期 ________ 停留期間 ___________________

________________________日期 ________ 停留期間 ___________________

________________________日期 ________ 停留期間 ___________________

8. 在湖北期間是否有曾接觸活家禽？ □有 □沒有
9. 在湖北期間是否有曾食用野味（如果子狸、蝙蝠等）？ □有 □沒有

E. 疫情期間採取個人防護措施情況

1. 從哪裡獲得防護知識？

   □ 電視 □ 電台 □ 報章 □ 社交媒體(如微信)
2. 疫情期間主要配戴哪種口罩？

   □ 布/防塵口罩 □ 外科口罩 □ N95 或以上級別口罩
3. 在湖北期間外出都會配戴口罩？ □每次 □偶爾 □不會
4. 在湖北期間與人(同住親人除外)接觸/交談都會配戴口罩？

   □每次 □偶爾 □不會
5. 你認為配戴口罩後，手意外接觸口鼻的次數? □減少 □不變 □增加
6. 你是否認同配戴口罩後，勤洗手便不再重要? □是 □否
7. 在湖北疫情期間是否會經常用肥皂或梘液洗手？ □是 □否
8. 在湖北疫情期間，如現場沒有洗手設施，是否會以酒精搓手液或消毒濕紙巾潔手？ □是 □否
9. 在湖北疫情期間是否有定期對家居進行清潔消毒(例如使用 1:99 漂白水或其他消毒產品)？ □有 □沒有

   如有，進行清潔消毒的頻率：

   □每天 1 次 □每 2-3 天 1 次 □每星期或以上 1 次
10. 在湖北疫情期間是否有定時清潔消毒手機？ □有 □沒有
11. 在湖北疫情期間，外出回家後是否會即時對衣物或隨身物品進行清潔消毒？

    □是 □否
12. 疫情期間，在湖北是否有參與聚餐(同住家人共餐除外)？ □有 □沒有

    如有，共聚餐的次數：□1-2 次 □3-5 次 □5 次以上

    在湖北平均每月聚餐次數：__________次

共餐的對象(可多選)：□親人　□鄰居　□朋友　□同事　□其他__________

有否曾參加「百步亭」宴會？　□有　□沒有

13. 疫情期間，飲食習慣是否有以下改變（可多選）:

□多喝水　□多吃蔬菜水果　□少吃煎炸食品

F. 疫情以前在澳門採取個人防護措施情況

1. 以往在澳門外出都會配戴口罩？　□會　□不會
2. 以往在澳門與人(同住親人除外)接觸/交談都會配戴口罩？
   □每次　□偶爾　□不會
3. 以往在澳門是否會經常用肥皂或梘液洗手？　□是　□否
4. 以往在澳門，如現場沒有洗手設施，是否會以酒精搓手液或消毒濕紙巾潔手？　□是　□否
5. 以往在澳門是否有定期對家居進行清潔消毒(例如使用 1:99 漂白水或其他消毒產品)？　□有　□沒有
   如有，進行清潔消毒的頻率：
   □每天 1 次　□每 2-3 天 1 次　□每星期或以上 1 次
6. 以往在澳門是否有定時清潔消毒手機？　□有　□沒有
7. 以往在澳門，外出回家後是否會即時對衣物或隨身物品進行清潔消毒？
   □是　□否
8. 以往在澳門是否有參與聚餐(同住家人共餐除外)？　□有　□沒有
   如有，在澳門平均每月聚餐次數：__________次
   共餐的對象(可多選)：□親人　□鄰居　□朋友　□同事　□其他__________

以下由醫務人員填寫

身高：____________　體重：____________

慢性病：__________________________________
