# Supplementary material for: The common personal behavior and preventive measures among 42 uninfected travelers from the Hubei province, China during COVID-19 outbreak: a cross-sectional survey in Macao SAR, China
Source: PeerJ. 2020 Jun 19;8:e9428. doi: 10.7717/peerj.9428 (PMC7307564; doi:10.7717/peerj.9428)
Supplement: Supplemental Information 3 [file peerj-08-9428-s003.pdf]

# Questionnaire Survey for travelers returned from Hubei province

**Year/Month/Date**

**(English Translation)**

## A. Personal Information

Name:______________ Gender: □ Male □ Female Date of Birth: __________________

Nationality:________ Telephone:__________________ Occupation: ___________________

Education level: □ Primary education □ Secondary education

□ Bachelor's degree □ Master's degree or above

Address in Hubei:

______________________________________________________________________________

## B. Personal health conditions and habits:

1. What is the most important reason(s) of not getting COVID-19 in your opinion? (Can select more than one option):
   □ Good health condition
   □ Good personal protective measures
   □ Stay away from the crowd / Decrease cluster or gathering incidence
   □ Relatively safe in the community
   □ Diet and lifestyle (Balanced diet 、Physical training)

2. During Hubei outbreak, did you take any medications/health products to prevent the disease?
   □ Yes, please tick the following:
      □ Medicine, please specify the name: ________________________________
      □ Herbal medicine, please specify the name: _______________________
      □ Health products (Dietary supplements /Vitamins, etc.)
      Please specify the name: ________________________
   □ No

3. What was your mood when you were in Hubei?
□ Nervous □ Anxious □Worried □Calm
Other, please specify: ____________________________________________

4. Do you have any chronic disease(s)？
□ Yes, please tick the follow option(s):
□ Hypertension □ Coronary artery disease □ Diabetes Mellitus
□ Dyslipidemia □ Gout/Hyperuricemia
□ Cerebrovascular disease (Stroke) □ Chronic kidney disease
□ Hepatitis □ Cirrhosis
□ Chronic obstructive pulmonary disease
□ Other, please specify: ______________________________
□ No

5. Do you have the habit of chronic medications use？
□ Yes, please specify: ____________________________________________

□ No

6. Do you have the habit of chronic herbal medicine use？
□ Yes, please specify: ____________________________________________

□ No

7. What is your usual preference on choosing medicine / herbal medicine when you are sick？
□ Herbal medicine □ Medicine

8. During Hubei outbreak, please tick the following options regarding your health promotion behavior:
Doing sport: □ Less than before □ No change □ More than before
Enough sleep: □ Less than before □ No change □ More than before
Drink more water: □ Less than before □ No change □ More than before
Buy mask and disinfectants: □ Less than before □ No change □ More than before
Wash hands: □ Less than before □ No change □ More than before
Search for health information: □ Less than before □ No change □ More than before

9. Do you have the following symptom/signs during Hubei outbreak:
   - □ Fever Start _____ End______ □ Cough Start _____ End______
   - □ Weakness Start _____ End______ □ Dyspnea Start _____ End______
   - □ Sore-throat Start _____ End______ □ Runny nose Start _____ End______
   - □ Pneumonia Start _____ End______ □ Abdominal pain Start _____ End______
   - □ Vomit Start _____ End______ □ Diarrhea Start _____ End______

   Others, please specify: ____________________________________________________

10. Do you have regular physical activities (including work, housework and sports)?
    - □ Housework: Sweeping, cleaning, etc., walking
      Average per week: □less than 1 hour □1-3hours □more than 3 hours
    - □ Jogging、Riding bicycles、Playing Tai chi
      Average per week: □less than 1 hour □1-3hours □more than 3 hours
    - □ Ball games related competitive sports
      Average per week: □less than 1 hour □1-3hours □more than 3 hours
    - □ No

11. Do you smoke? □Current smoker □Ex-smoker □Never

12. Do you have alcoholic abuse? □Yes □No

**C. Schedule and Routine in Hubei province**

1. Do you live/stay in Hubei for long term? □Yes □No

   If no, duration of stay in Hubei:__________to___________

   Reason for visit Hubei： □ Visit relatives □Business trip
   □ Other reasons _________________

2. Accommodation type:

   □ Hotel □ House □ House for rent

3. Location of accommodation:

   □ City center □ Countryside or town □ Village

4. Number of people cohabit with: ____________ people
5. Area of residence: _______________________square feet
6. Number of people cohabit to share the bedroom:

   □ Alone □ 2 people □ 3 people □ 4 people or above

7. Besides the above written address, did you visit other cities in Hubei province ? □ Yes □ No

   If yes, please specify:

   i. Name of city_____________ Days of stay__________________________

      Reason__________________ Means of transport_____________________

   ii. Name of city_____________ Days of stay__________________________

      Reason__________________ Means of transport_____________________

   iii. Name of city_____________ Days of stay__________________________

      Reason__________________ Means of transport_____________________

8. During your stay in Hubei, did you visit other provinces besides Hubei? □Yes □No

   If yes, please specify:

   i. Name of province_____________ Days of stay_____________________

      Reason______________________ Means of transport________________

   ii. Name of province_____________ Days of stay_____________________

      Reason______________________ Means of transport________________

   iii. Name of province_____________ Days of stay_____________________

      Reason______________________ Means of transport________________

9. Daily life during stay in Hubei:

   □ Buy daily commodities by yourself

   □ Receive daily supply via the unified delivery

10. Did you receive phone call from your clinic during stay in Hubei?

   □ Yes □No

**D. Contact History**

1. During stay in Hubei, who were the people you have contact with mostly?

   □ Family □ Friends □ Colleagues □ Others___________________________

2. During stay in Hubei, did you go to any hospital or clinic? □Yes □No

   If yes, reason of go to hospital or clinic: _________________________

   Date and duration of stay: ___________________________________

3. During stay in Hubei, did you have any admission history in hospital? □Yes □No

   If yes, reason of admission, name of hospital and duration of stay:

   ___________________________________

   People you have contact with mostly during admission: □ Family □ Friends □ Working staff

4. During stay in Hubei, did you have contact with suspected/confirmed COVID-19 patients?

   □ Yes □No

   If yes, way of contact (e.g. having meal): ________________________________

   Duration of contact: □10min or less □10-30min □30min or more

5. During stay in Hubei, did your family have contact with suspected/confirmed COVID-19 patients？

   □Yes □No

   If yes, way of contact (e.g. having meal):_______________________________

   Duration of contact: □10min or less □10-30min □30min or more

6. Were there any confirmed COVID-19 cases in the local community you stayed in Hubei?

   □Yes □No

   If yes, please specify: __________________________________________

7. Between 01/01 to “lockdown” period, did you do the following:

   Use of public transportation □No □ Yes Specify:

Go to supermarket Date____________ Duration of stay ______________

Go to traditional market (wet market) Date____________ Duration of stay ______________

Visit crowded places:

___________________________________ Date _____________ Duration of stay_____________

___________________________________ Date _____________ Duration of stay _____________

___________________________________ Date _____________ Duration of stay_____________

___________________________________ Date _____________ Duration of stay_____________

8. During stay in Hubei, did you have any contact with live poultry? □Yes □No
9. During stay in Hubei, have you eaten any game meats (e.g. civets, bats, etc.)? □Yes □No

**E. Personal preventive measures conditions during the outbreak**

1. Where did you receive the information of prevention？

   □ Television □ Radio □ Newspapers □ Social media (e.g. “Wechat”)

2. Which kind of mask you mostly use during the outbreak?

   □ Cloth/Dust mask □ Surgical mask □ N95 or above grade respirator

3. Did you wear a mask when outdoor during the Hubei outbreak？

   □Every time □Occasional □No

4. Did you wear a mask when contact/talk with people (except those living together) during the Hubei outbreak？ □Every time □Occasional □No
5. What do you think about the frequency of accidental face or nose touching after wearing a mask? □Decrease □Unchange □Increase
6. Do you think that it is less important to wash hands after wearing a mask? □ Yes □No
7. During Hubei outbreak, did you often wash your hands with soap/liquid soap?

   □Yes □No

8. During Hubei outbreak, if there was no handwashing facility on-site, would you wash your hands with alcohol-based hand sanitizers or disinfected wipes? □Yes □No
9. During Hubei outbreak, did you clean and disinfect your house regularly (e.g., using 1:99 bleach water or other disinfectants)? □Yes □No

   If yes, frequency of household cleaning and disinfection:

   □Once a day □Once every 2-3 days □Once a week or more than a week
10. During Hubei outbreak, did you clean mobile phone regularly? □Yes □No
11. During Hubei outbreak, did you clean clothes and personal belongings immediately once you get back home? □Yes □No
12. During Hubei outbreak, did you attend any meal gatherings (except for family members cohabit with) regularly? □Yes □No

    If yes, total number of gatherings: □1-2 times □3-5times □ > 5times

    Number of meal gatherings per month in Hubei: ___________times

    People you had meal with (Can choose more than one option):

    □Relatives □Neighbors □Friends □Colleagues □Others___________

    Have you joined the "Bai Bu Ting" banquet? □Yes □No
13. During Hubei outbreak, did you have the following change in diet habit? (Can choose more than one option):

    □Drink more water □Eat more vegetables and fruits □Less fried food

## F. Personal preventive measures conditions in Macau before the outbreak

1. When you were in Macau before outbreak, did you wear a mask every time when outdoor?

   □Yes □No
2. When you were in Macau before outbreak, did you wear a mask when contact/talk with people (except those living together)?

   □Every time □Occasional □No

3. When you were in Macau before outbreak, did you often wash your hands with soap/liquid soap? □Yes □No
4. When you were in Macau before outbreak, if there was no handwashing facility on-site, would you wash your hands with alcohol-based hand sanitizers or disinfected wipes? □Yes □No
5. When you were in Macau before outbreak, did you clean and disinfect your house regularly (e.g., using 1:99 bleaching water or other disinfectants)? □Yes □No

   If yes, frequency of household cleaning and disinfection:

   □Once a day □Once every 2-3 days □ Once a week or more than a week
6. When you were in Macau before outbreak, did you clean mobile phone regularly? □Yes □No
7. When you were in Macau before outbreak, did you clean clothes and personal belongings immediately once you get back home? □Yes □No
8. When you were in Macau before outbreak, did you attend any meal gatherings (except for family members cohabit with) regularly? □Yes □No

   If yes, number of meal gatherings per month in Macao: __________times

   People you had meal with (Can choose more than one option):

   □Relatives □Neighbors □Friends □Colleagues □Others___________

--------------------------------------------------------------------------------

The following will be filled in by medical staff

Body Height: _____________ Body weight: _____________

Chronic diseases: ______________________________________
